# A cetacean-specific CNE suppresses osteogenesis to drive appendicular modification in secondary aquatic adaptation

**DOI:** 10.1016/j.isci.2026.117145

**Published:** 2026-09-03

**Authors:** Yao Liu, Zhenhua Zhang, Fenli Zhang, Shixia Xu, Guang Yang

**Affiliations:** 1 Jiangsu Key Laboratory for Biodiversity and Biotechnology, College of Life Sciences, Nanjing Normal University, Nanjing, Jiangsu 210023, China; 2 Southern Marine Science and Engineering Guangdong Laboratory (Guangzhou), Guangzhou, Guangdong 511458, China

**Keywords:** cetaceans, CNE300, mouse model, reduced osteogenesis, skeletal alteration

## Abstract

Cetaceans represent a hallmark of secondary aquatic adaptation, accompanied by extensive appendicular remodeling, whose underlying regulatory mechanisms remain challenging to resolve. Here, we investigated a cetacean-specific conserved non-coding element, CNE300, which exhibits accelerated evolution and reduced regulatory activity in cell-based assays. Homozygous cetacean CNE300 knock-in mice showed significantly shortened appendicular long bones and trabecular microarchitectural alterations, including reduced trabecular density. These phenotypic changes were concordant with key features of cetacean limb evolution. Transcriptomic profiling of embryonic limb buds identified a distinctive transcriptional bias toward cartilage differentiation programs in limb developmental genes, and postnatal endochondral ossification exhibited reduced osteogenic activity with normal bone resorption, suggesting that reduced osteogenesis might be a major contributor to the skeletal alterations observed in knock-in mice and natural cetacean limbs. Our findings demonstrated that CNE300 functions as a regulatory element driving appendicular remodeling and provide direct mechanistic insight into the molecular basis underlying cetacean limb evolution.

## Highlights

• Cetacean-specific CNE300 regulates appendicular skeletal development
• Cetacean CNE300 shortens limb long bones and reduces trabecular bone mass
• CNE300 may contribute to appendicular remodeling in cetaceans through suppressed osteogenesis

## Graphical abstract

**Figure:**
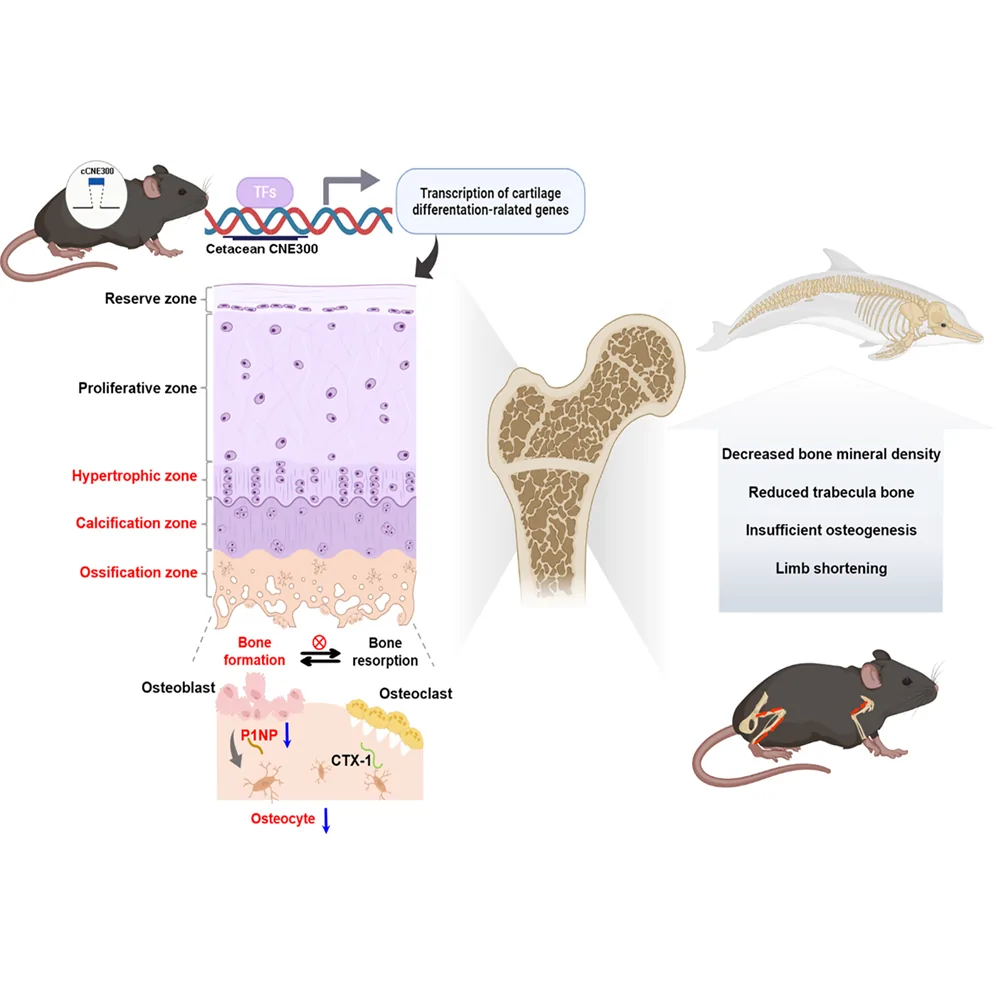

## Teaser

Zoology; Molecular biology; Evolutionary biology; Evolutionary mechanisms

## Introduction

Appendages represent one of the most morphologically diverse and functionally differentiated structures in the evolutionary history of vertebrates, serving as a hallmark of adaptive radiation and ecological niche expansion.^1^^,^^2^^,^^3^ From the primitive fins of Devonian sarcopterygian fishes to the diverse limb architectures of tetrapod, shifts in appendicular morphology have fundamentally shaped animals’ capacity for locomotion, resource acquisition strategies, and ecological niche exploitation.^4^^,^^5^ For example, cetaceans, as a lineage that secondarily returned to the aquatic environment, have undergone pronounced and directional limb remodeling, with forelimbs transformed into flippers for maneuvering and hindlimbs regressed or lost to reduce locomotor costs.^6^^,^^7^^,^^8^ The extreme adaptive remodeling of cetacean appendicular morphology provides an informative model for investigating the mechanisms underlying morphological diversification and functional adaptation.

Previous studies on cetacean limb evolution primarily focused on the evolutionary patterns and functional analyses of protein-coding genes involved in limb development, such as the *Homeobox* (*Hox*) family members and some key signaling pathways (e.g., Bone morphogenetic protein [BMP]; Fibroblast growth factor [FGF]; Sonic hedgehog [SHH]; and Wingless/INT [WNT]).^9^^,^^10^^,^^11^ Although these investigations have provided important insights into the molecular basis of cetacean limb evolution, they have largely been constrained due to the high evolutionary conservation of protein-coding genes involved in limb development. By contrast, recent studies increasingly suggested that non-coding regulatory elements, particularly conserved non-coding elements (CNEs), may play a more important role in driving lineage-specific diversification of appendicular phenotypes.^12^^,^^13^

CNEs act as central regulatory elements that modulate the spatiotemporal expression pattern of genes during development and contribute to phenotypic remodeling and evolution.^14^^,^^15^ Functional divergence of CNEs has been linked to major evolutionary transitions across vertebrates, highlighting their central role in connecting regulatory evolution with complex phenotypic diversification.^16^^,^^17^^,^^18^ However, although recent comparative genomics analyses have identified a series of cetacean limb-associated CNEs, only weak functional correlations or mechanistic links between these CNEs and cetacean limb changes have been established because such CNEs did not lead to significant appendicular phenotypic alterations in transgenic mouse models, possibly due to functional redundancy or compensation of additional enhancers in the mouse genome.^19^^,^^20^

Here, we focused on CNE300, a cetacean-specific CNE characterized with accelerated evolution and reduced regulatory activity at the cellular level.^21^ A cetacean CNE300 knock-in mouse model was constructed using the CRISPR/Cas9 technique, and the homozygous knock-in mice displayed significant alterations of limb skeletal elements that were congruent with appendicular changes in cetaceans. Transcriptomic, histological, and bone remodeling-associated assessments supported that reduced osteogenic activity represented a major contributor to the skeletal changes observed in limbs. Our findings provided robust causal evidence for CNE300-mediated regulatory modulation in appendicular remodeling and highlighted the broader role of CNEs in driving lineage-specific phenotypic diversification.

## Results

### Construction of a cetacean CNE300 knock-in mouse model

A knock-in mouse model was generated in which the endogenous locus was replaced with the cetacean CNE300 sequence using CRISPR/Cas9-mediated genome editing (Figure S1A). Genomic DNA was isolated from offspring obtained through natural breeding of F1 heterozygous mice, and genotyping was performed by using PCR with custom-designed primers. Sanger sequencing confirmed the presence of homozygous animals carrying the cetacean-specific 11-bp deletion within CNE300 (Figures S1B and S1C). The homozygous line was expanded through controlled breeding to obtain sufficient individuals for subsequent phenotypic and molecular analyses.

### Cetacean CNE300 homozygous knock-in mice exhibit shortening of limb long bones

**Figure 1:**
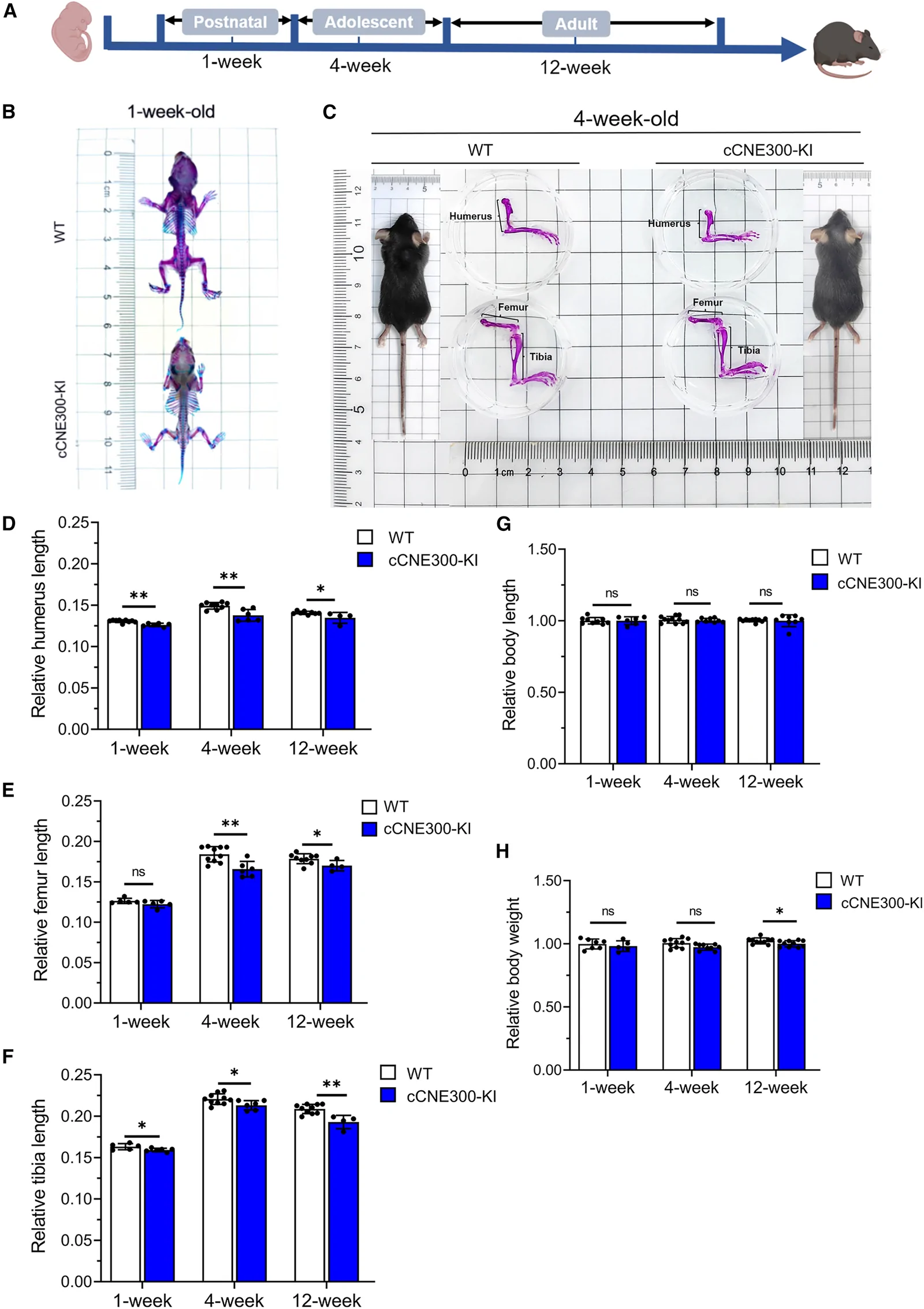
Morphometric analysis of WT and cCNE300-KI mice limb skeletons at different developmental stages (A) Schematic diagram showing the histomorphometry analysis of mice at different ages. (B) Representative Alcian blue/Alizarin red staining images of the whole skeleton of 1-week-old WT and cCNE300-KI mice. (C) Representative Alizarin Red staining images of the whole skeleton of 4-week-old female WT and cCNE300-KI mice, alongside their isolated humerus, femur, and tibia. (D–F) Quantification of the relative lengths of humerus (D), femur (E), and tibia (F) at the indicated ages. Relative bone lengths were calculated by normalizing the absolute bone length of each individual mouse to its own corresponding body length. (G) Quantification of the relative body length of WT and cCNE300-KI mice at different ages. (H) Relative body weight of WT and cCNE300-KI mice at different ages. In the bar charts, each dot represents an individual sample (*n* ≥ 4). Data are presented as mean ± SD. Student’s *t* test, *∗p* < 0.05; *∗∗p* < 0.01; ns, not significant. See also Table S1.

Morphometric measurements were performed at three critical developmental time points (1-week, 4-week, and 12-week, representing early postnatal, adolescent, and adult periods) in mice (Figure 1A). Alcian blue and Alizarin red double staining showed that cCNE300-KI mice exhibited significantly shortened limb long bones of the limbs beginning as early as 1 week after birth (Figures 1B–1F; Table S1). To further examine the status of the most distal bones, we performed morphological observations of the forelimb and hindlimb autopods. The metacarpals, metatarsals, and phalanges displayed normal structural integrity and patterning in cCNE300-KI mice, comparable with WT controls (Figure S2). Under standard chow (STC) feeding conditions, cCNE300-KI mice showed no significant difference in body length compared with WT (Figure 1G; Table S1), but exhibited a slightly lower body weight at 12 weeks (KI-mice: 1.000 ± 0.025; WT: 1.023 ± 0.023; *p* = 0.0452, Figure 1H; Table S1).

### Cetacean CNE300 alters limb skeletal microarchitecture and reduces trabecular bone mass

**Figure 2:**
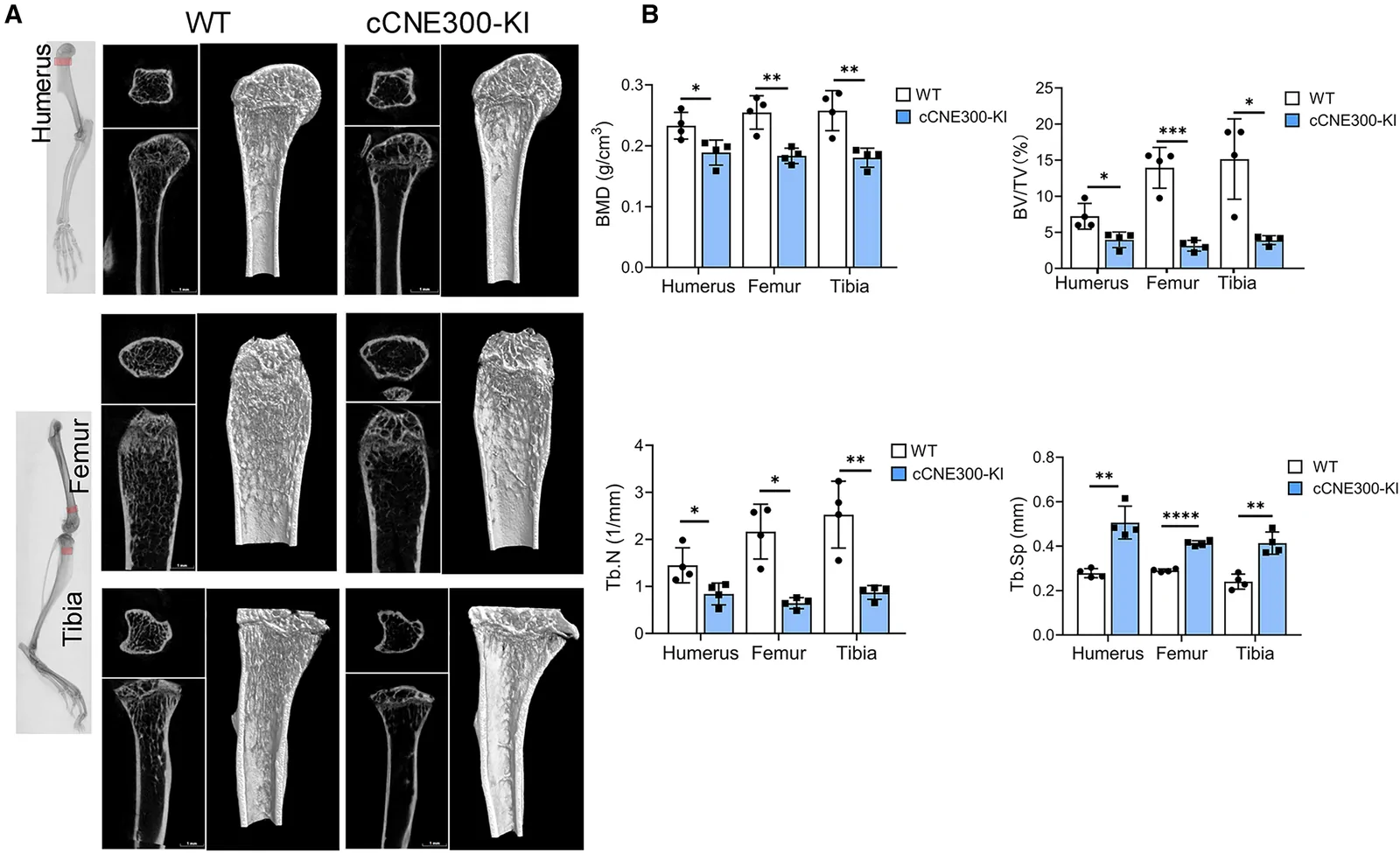
Micro-CT analysis reveals reduced trabecular bone mass in cCNE300-KI mice (A) Representative micro-CT images showing 2D cross-sectional views and 3D reconstructions of the proximal humerus, distal femur, and proximal tibia from 12-week-old female WT and cCNE300-KI mice. Scale bars, 1 mm. (B) Quantitative analyses of trabecular bone parameters, including bone mineral density (BMD), bone volume fraction (BV/TV), trabecular number (Tb.N), and trabecular separation (Tb.Sp). In the bar charts, each dot represents one appendicular bone sample from an individual mouse (*n* = 4). Data are presented as mean ± SD. Student’s *t* test, *∗p* < 0.05; *∗∗p* < 0.01; *∗∗∗p* < 0.001; *∗∗∗∗p* < 0.0001. See also Table S2.

**Figure 3:**
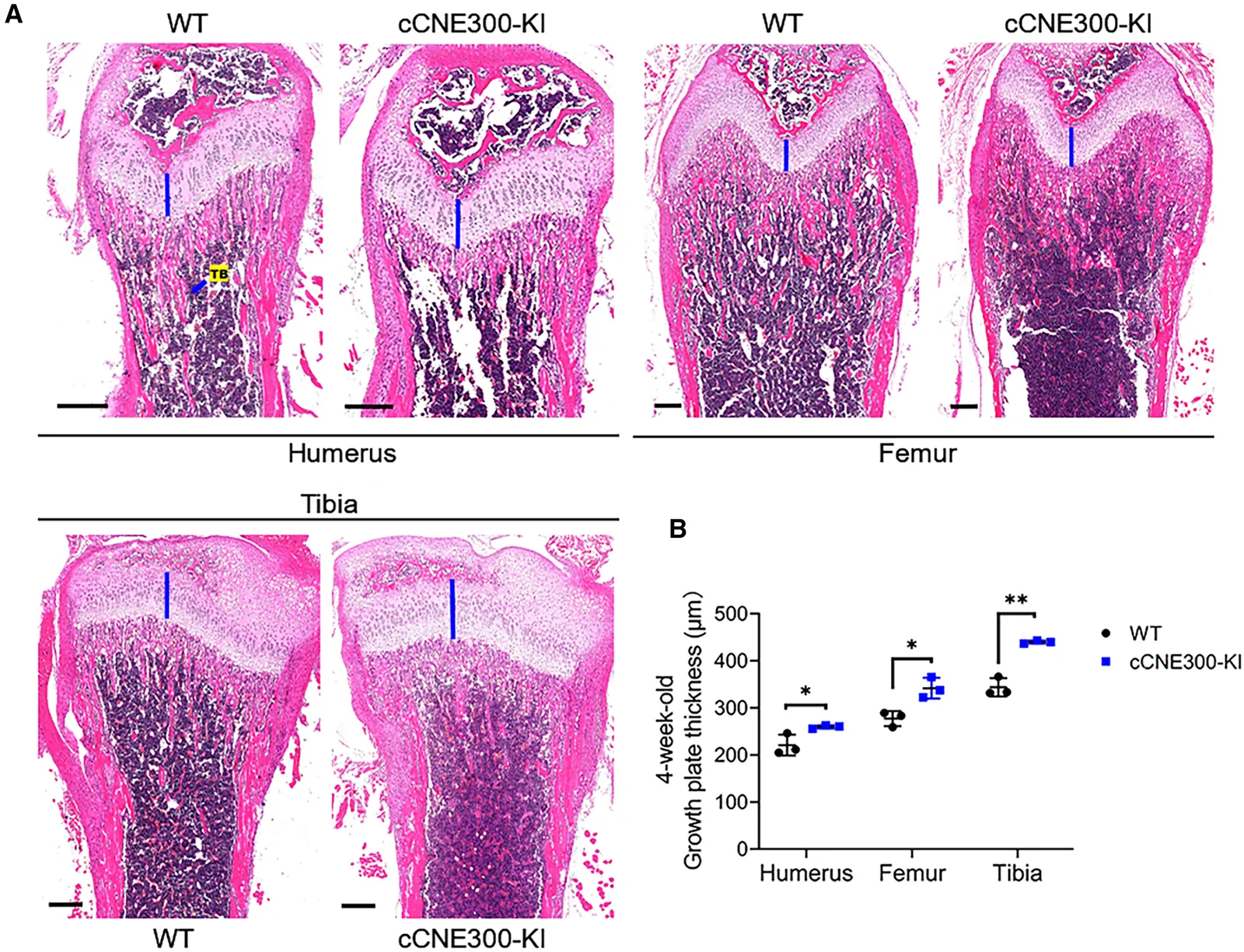
Representative images of H&E staining of limb bone sections of WT and cCNE300-KI mice with the indicated ages (A) Representative H&E staining of longitudinal sections of the humerus, femur, and tibia from 4-week-old female WT and cCNE300-KI mice. Blue arrowheads and blue line segments indicate trabecular bone (TB) and the growth plate, respectively. Scale bars, 250 μm. (B) Quantification of the growth plate thickness of the indicated mice, each dot represents one appendicular bone sample from an individual mouse (*n* = 3). Data are presented as mean ± SD. Student’s *t* test, ∗*p* < 0.05, ∗∗*p* < 0.01. See also Figure S4.

To evaluate the bone microarchitecture, micro-CT analysis was performed on limb long bones, including the humerus, femur, and tibia. Compared with WT, 12-week-old cCNE300-KI mice exhibited a sparser reticular trabecular structure in the long bones. Quantitative analysis revealed significant reductions in bone mineral density (BMD; humerus: *p* = 0.026; femur: *p* = 0.003; and tibia: *p* = 0.005), bone volume fraction (BV/TV; humerus: *p* = 0.020; femur: *p* < 0.001; and tibia: *p* = 0.029), and trabecular number (Tb.N; humerus: *p* = 0.032; femur: *p* = 0.012; and tibia: *p* = 0.029), accompanied with a significant increase in trabecular separation (Tb.Sp; humerus: *p* = 0.001; femur: *p* < 0.0001; and tibia: *p* = 0.001) (Figures 2A and 2B; Table S2). To assess whether these skeletal alterations were systemic, we additionally scanned and analyzed the skull and spine (L4-L5), but no significant differences were detected in skull or spine measurements between cCNE300-KI and WT mice (Figure S3; Table S3), suggesting that the observed skeletal phenotypes are unlikely to reflect systemic alterations in bone development. Additionally, consistent with micro-CT analyses, H&E staining revealed a markedly sparser trabecular structure in cCNE300-KI mice than that in WT (Figure S4). Furthermore, growth plate thickness was significantly increased in cCNE300-KI mice compared with WT controls at both 4 and 12 weeks of age (Figures 3 and S4).

### Cetacean CNE300 induces transcriptomic alterations in limb buds

**Figure 4:**
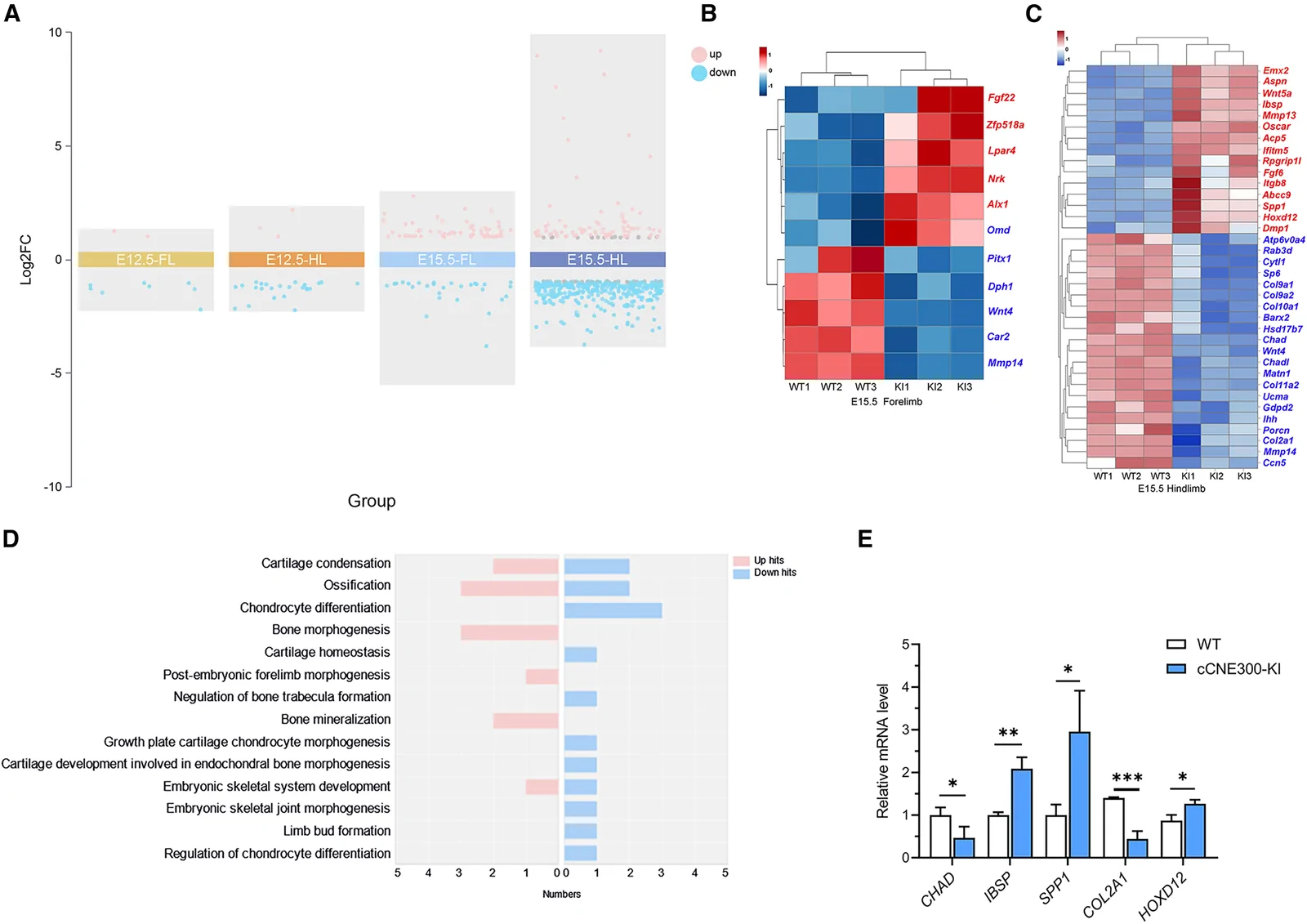
RNA-Seq analysis of forelimb and hindlimb buds at different developmental stages of WT and cCNE300-KI mice (A) The DEG counts in the mice forelimb and hindlimb at E12.5 and E15.5. DEGs are indicated by points (red, up-regulated; blue, down-regulated). (B) Hierarchical clustering of limb development-associated DEGs in Forelimb at E15.5. (C) Hierarchical clustering of limb development-associated DEGs in Hindlimb at E15.5. (D) GO functional clustering of key candidate limb-related DEGs in biological processes. (E) Relative mRNA expression levels of selected candidate DEGs in limb bud at E15.5 (*n* = 3 independent biological replicates for each group). Data are presented as mean ± SD. Student’s *t* test, *∗p* < 0.05; *∗∗p* < 0.01; *∗∗∗p* < 0.001. See also Tables S4 and S5.

Transcriptomic profiling of embryonic limb buds across key developmental stages showed a processive increase in the number of downregulated differentially expressed genes (DEGs) as limb development progressed (Figure 4A). At E12.5, relatively few DEGs were identified in limb buds of cCNE300-KI mice embryos (Table S4), with functional enrichment primarily associated with extracellular matrix-related processes in the forelimb and muscle-related processes in the hindlimb (Figure S5). In contrast, cCNE300-KI mice embryos displayed significant differential expression of key limb development genes at E15.5 compared with WT controls (Table S5). Functional enrichment analysis indicated that DEGs were mainly associated with biological processes involved in endochondral ossification, including cartilage differentiation and regulation (e.g., *Col2a1*, *Matn1*, *Cytl1*, *Chadl*, *Chad*, and *Wnt5a*) and bone mineralization (e.g., *Hoxd12*, *Wnt4*, *Oscar*, *Aspn*, *Acp5*, and *Mmp13*) (Figures 4B–4D). qPCR validation of candidate DEGs confirmed the expression patterns identified in the RNA-seq results (Figure 4E).

### Cetacean CNE300 suppresses osteoblast-mediated bone formation without impacting osteoclast activity

**Figure 5:**
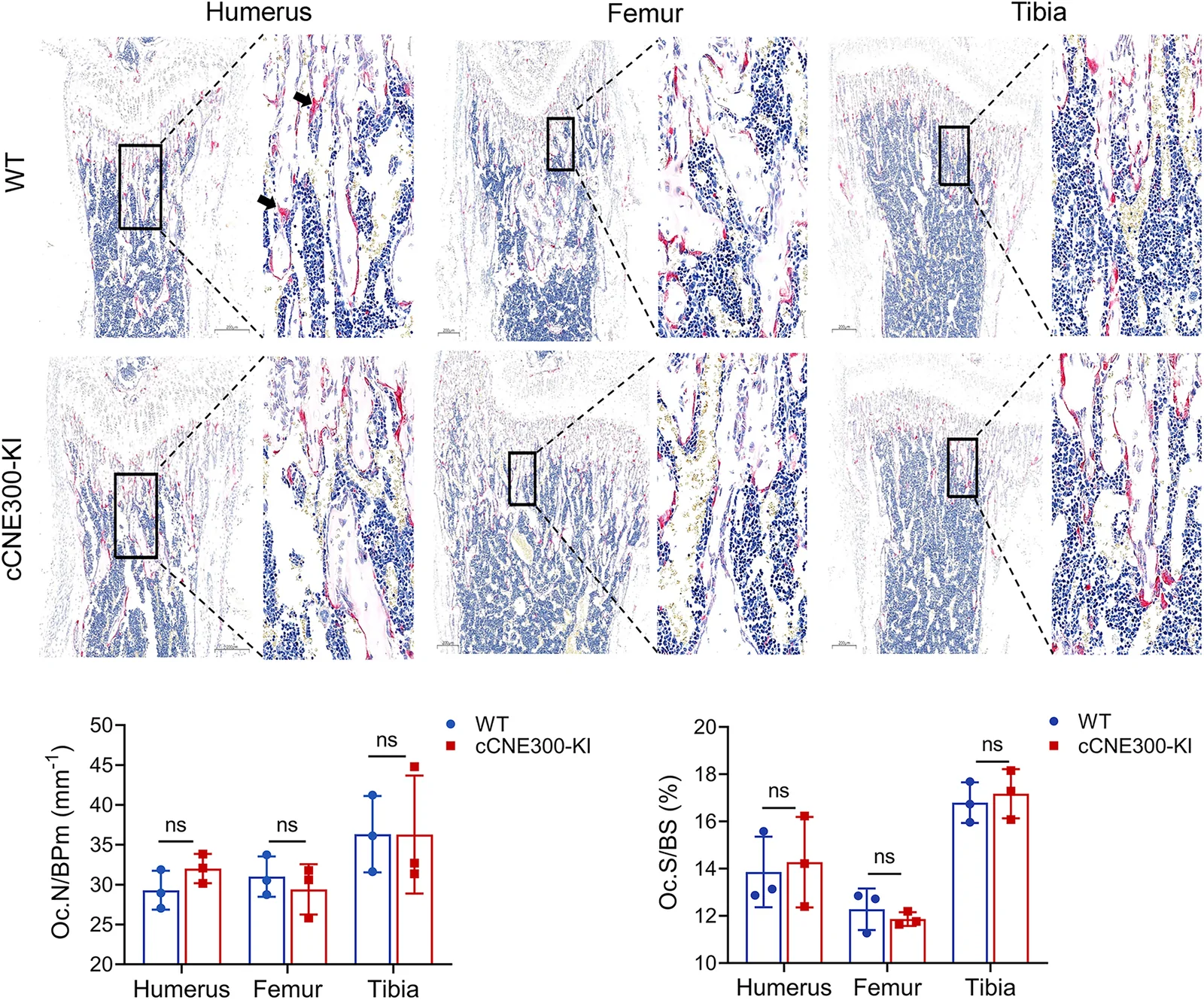
Representative image of TRAP staining limb bone sections of female WT and cCNE300-KI mice The right images depict the enlarged frames in the left images. Scale bars, 200 μm. The positively stained cells are denoted by the black arrows (upper images). Quantification of the osteoclast number/bone perimeter (Oc. N/BP) and Osteoclast surface/bone surface (Oc. S/BS) (lower images). In the bar charts, each dot represents one appendicular bone sample from an individual mouse (*n* = 3). Data are presented as mean ± SD, Student’s *t* test, ns, not significant.

**Figure 6:**
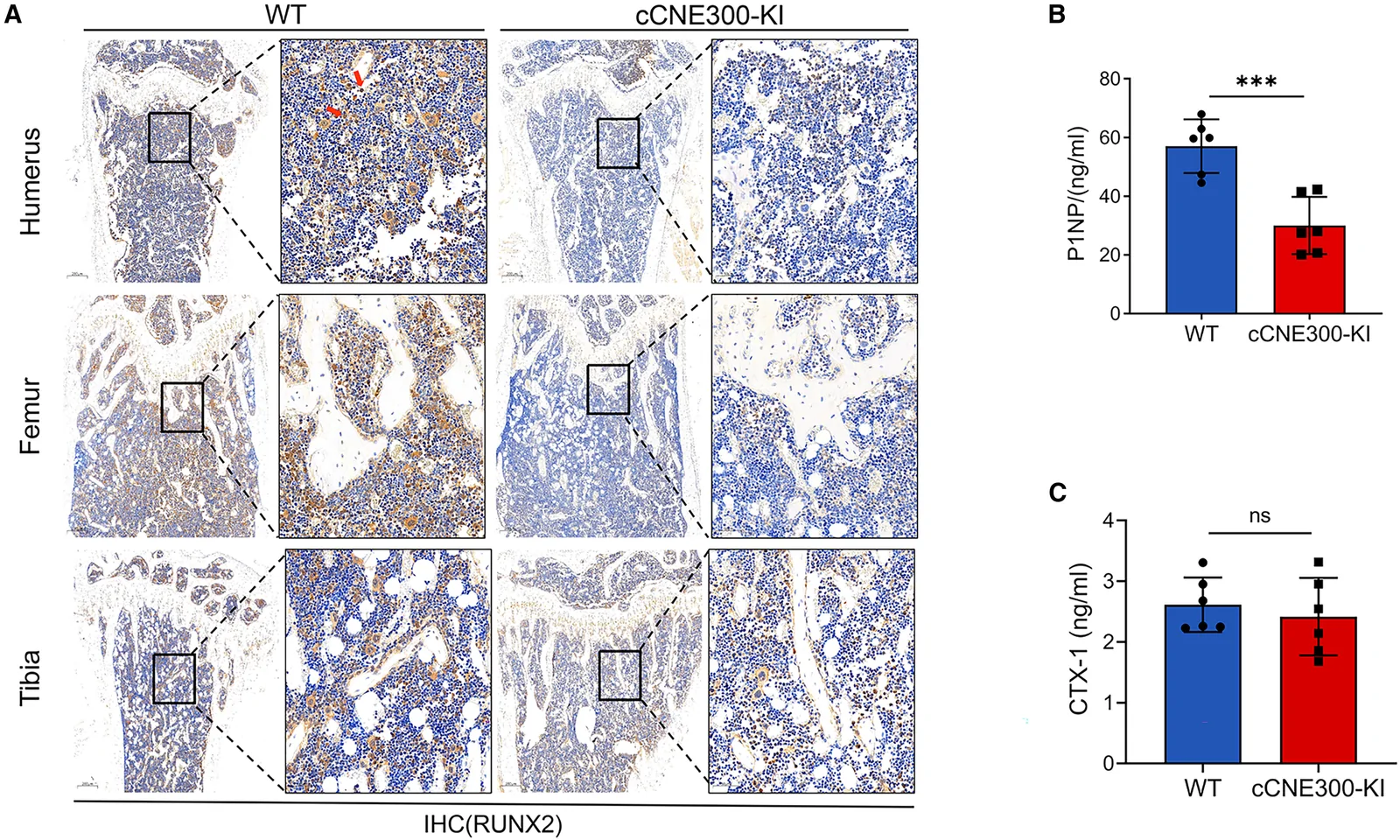
Immunohistochemical and osteogenic activity analyses (A) Immunohistochemical IHC staining for RUNX2 of bone sections. The right images depict the enlarged frames in the left images. Scale bars, 200 μm. The positively stained cells in brown are indicated by red arrows. (B) ELISA measurements of P1NP. (C) CTX-1 from 12-week-old female WT and cCNE300-KI mice (*n* = 6 sample for each group). Results are expressed as mean ± SD, Student’s *t* test, *∗∗∗p* < 0.001; ns, not significant.

To further investigate the potential mechanisms underlying the microarchitectural alterations and reduced bone mass observed in cCNE300-KI mice, we examined the functional activity of osteoblasts and osteoclasts in the limb long bones. TRAP staining showed no significant differences in either the number or surface area of TRAP-positive osteoclasts of the two groups (Figure 5), whereas immunohistochemical analysis revealed a marked reduction in nuclear localization of RUNX2 in cCNE300-KI mice (Figure 6A). Subsequent serum biochemical assays showed a significantly lower level of the bone formation marker P1NP (Figure 6B), whereas the bone resorption marker CTX-1 remained unchanged (Figure 6C).

## Discussion

Cetaceans have undergone a profound evolutionary transition from terrestrial to fully aquatic environments, with appendicular remodeling representing one of the most prominent morphological transformations associated with secondary aquatic adaptation.^22^ Although CNEs have been increasingly recognized as key regulators of morphological innovation, including limb development and modification of animals, very few functional studies have been conducted, and the regulatory mechanisms underlying appendicular remodeling during secondary aquatic adaptation in cetaceans have not been adequately resolved so far.^19^^,^^20^^,^^21^ For example, recent functional analyses of limb-associated CNEs, such as *Tbx4*-HLEA and *Gli3*-hs1586, suggested their regulatory involvement during early limb development in mouse models; however, no obvious appendicular phenotypes were produced *in vivo* and could not provide direct functional evidence to link specific CNEs to limb modification.^19^^,^^20^^,^^23^

By contrast, in the present study, functional examination of a cetacean-specific CNE300 using a knock-in mouse model revealed significant appendicular skeletal phenotype changes, including shortened long bones and reduced trabecular density (Figures 1 and 2). It was well known that cetacean appendages have undergone adaptive modifications during the transition to aquatic environments, accompanied with extensive skeletal specialization, such as the progressive reduction of hindlimbs and relative decrease in bone density.^6^^,^^24^^,^^25^ Thus, the appendicular phenotype changes observed in the mouse model were partially consistent with those major features of limb skeletal remodeling in cetaceans, suggesting that cetacean-specific CNE300 may have played an important role in driving appendicular changes in cetaceans.

RNA-Seq analysis of homozygous KI mice limb buds revealed a widespread downregulation of DEGs, with a transcriptional bias toward programs associated with chondrogenic differentiation and endochondral ossification (Figure 4). This regulatory shift is to some extent consistent with the previously studied gene expression patterns in cetacean limb development,^8^^,^^26^^,^^27^ suggesting that divergence of CNE300 may alter the developmental trajectories of gene expression during initial limb bud formation. Furthermore, postnatal histological and biochemical analyses demonstrated that these transcriptional shifts further resulted in attenuated osteogenic activity, as evidenced by reduced nuclear localization of RUNX2, decreased serum P1NP levels, and persistent thickening of the growth plate in limb long bones (Figures 3, 6A, and 6B), collectively characterized a disruption of endochondral ossification. Notably, modulation of growth plate dynamics has been proposed as a key developmental mechanism underlying limb skeletal scaling and density variation in mammals.^28^^,^^29^ Previous studies have reported the specialized osteochondral adaptations in cetaceans (e.g., delayed secondary ossification centers, diminished calcified cartilage layer).^25^ These features were, at least partially, similar to those observed in KI mice, suggesting that insufficient bone formation might be one of the driving factors of the skeletal morphological alterations in cetaceans. Additionally, given that skeletal remodeling and homeostasis are governed by the dynamic balance between bone formation and resorption,^30^ the present analysis of bone resorption parameters, which revealed no significant changes in osteoclast activity or serum CTX-1 levels could exclude the principal contribution of osteoclast-mediated bone resorption to the observed skeletal phenotypes. In summary, our findings supported that divergence of cetacean CNE300 may preferentially disrupt osteoblast-mediated bone formation within the endochondral ossification program, thereby driving the skeletal shortening and reduced bone density during the evolutionary transformation of forelimbs into flipper or hindlimbs regression in cetaceans.

It was worth noting that, although the phenotypic alterations observed in this study are consistent with key morphological characteristics of cetacean limb evolution during secondary adaptation, the present KI mouse model did not fully recapitulate hallmark cetacean limb specializations, such as complete transformation of forelimbs into flippers or hindlimb regression.^6^ This observation suggests that coordinated appendicular remodeling during cetacean aquatic adaptation might require synergistic interactions among additional genetic, regulatory components, and biomechanical factors. In particular, the transition from terrestrial to aquatic environments markedly changes limb mechanical loading, which may further interact with developmental regulatory programs to shape bone growth and remodeling. The evolutionary history of appendicular bone density further supports this interpretation. Early fully aquatic archaeocetes, particularly basilosaurids, possessed markedly shortened hindlimbs, yet the femur remained very compact and osteosclerotic, likely serving buoyancy control during initial aquatic adaptation. In contrast, the forelimb elements exhibited a more spongious medullary organization, indicating that forelimbs and hindlimbs followed distinct microanatomical trajectories.^31^ In derived cetacean lineages, this regional heterogeneity gave way to a more generalized reduction in limb bone trabecular density, accompanied by highly modified appendicular elements that reflect further remodeling for locomotion.^24^^,^^25^ These contrasting patterns suggest that limb reduction and bone density changes in cetaceans were not a simple continuation of a single ancestral skeletal condition, but were progressively shaped across evolutionary stages and limb regions. In this context, the shortened long bones and reduced trabecular density observed in cCNE300-KI mice are more closely with the derived traits of more recent cetacean lineages, rather than with the osteosclerotic condition of early archaeocetes. This correspondence indicates that CNE300 may have contributed to a later-stage appendicular remodeling program superimposed upon earlier buoyancy-related skeletal adaptations. Its divergence thus represents one regulatory component within a broader stage- and region-specific evolutionary process, contributing to both limb shortening and reduced trabecular density through attenuated endochondral bone formation.

Additionally, the phenotypic effects induced by cetacean CNE300 (e.g., the decrease of bone density) were confined to the appendicular skeleton, which suggested that skeletal density decrease in other body regions is likely governed by different regulatory elements rather than a single and unified evolutionary mechanism. These findings collectively supported the complexity and diversity of regulatory mechanisms underlying phenotype evolution.^32^^,^^33^ Therefore, integration of more comprehensive genome-wide analyses and in-depth epigenomic evidence would be helpful to systematically identify additional key CNEs and functional gene sets in future, which are essential to better understand the molecular genetic basis underlying phenotypic change during secondary aquatic adaptation in cetaceans.

### Limitations of the study

Although the homozygous cetacean CNE300 knock-in mice exhibited key appendicular phenotypes associated with cetacean limb evolution, including shortened long bones and reduced trabecular density, the mouse model did not fully recapitulate hallmark cetacean limb specializations such as forelimb transformation into flippers or hindlimb regression. This limitation indicates that additional genetic, regulatory, and biomechanical factors are likely required to fully explain appendicular remodeling during secondary aquatic adaptation. In addition, the phenotypic effects of cetacean CNE300 were restricted to the appendicular skeleton, indicating that skeletal remodeling in other body regions is likely regulated by distinct molecular mechanisms. Future studies integrating genome-wide functional analyses and epigenomic evidence are needed to systematically identify additional regulatory elements and further elucidate the molecular basis of secondary aquatic adaptation in cetaceans.

## Resource availability

### Lead contact

All requests for additional information and resources should be directed to the lead contact, Guang Yang (gyang@njnu.edu.cn).

### Materials availability

All unique reagents and mouse strains generated in this study are available from the lead contact upon request.

### Data and code availability

• RNA-Seq data in this paper have been deposited in the NCBI Sequence Read Archive (SRA) BioProject: PRJNA14799650. All other data reported in this paper will be shared by the lead contact upon request.
• This study did not generate any new codes.
• Any additional information required to reanalyze the data reported in this paper will be shared by the lead contact upon request.

## Acknowledgments

We would like to thank the Jiangsu Key Laboratory for the Biodiversity Conservation and Sustainable Utilization in the Middle and Lower Reaches of the Yangtze River Basin, College of Life Sciences, Nanjing Normal University, for providing the facilities and contributions for this study. This work was financially supported by the 10.13039/501100012166National Key Research and Development Program, Ministry of Science and Technology (2022YFF1301600), the 10.13039/501100001809National Natural Science Foundation of China (U24A20362, 32030011), PI Project of Southern Marine Science and Engineering Guangdong Laboratory (GML2021GD0805), the Qinglan Project of Jiangsu Province, and the Priority Academic Program Development of Jiangsu Higher Education Institutions.

## Author contributions

G.Y. conceived the study and designed the experiments. Y.L. performed all sample collection, data analyses, and drafted the manuscript. Z.Z. and F.Z. participated in the analysis of the data. G.Y. and S.X. reviewed the manuscript. All authors read and approved the final manuscript version.

## Declaration of interests

The authors declare no competing interests.

## Declaration of generative AI and AI-assisted technologies in the writing process

During the preparation of this work, the authors used DeepSeek to improve readability and language. After using this tool, the authors reviewed and edited the content as needed and take full responsibility for the content of the published article.

## STAR★Methods

### Key resources table

**Table:** 

REAGENT or RESOURCE	SOURCE	IDENTIFIER
**Antibodies**		

Rabbit polyclonal anti-RUNX2	Abcolonal	Cat# A2851; RRID: AB_2764676
HRP-conjugated goat anti-rabbit IgG secondary antibody	Pinuofei Biotechnology	Cat# PN0046

**Chemicals, peptides, and recombinant proteins**		

Cas9 mRNA	GemPharmatech Co., Ltd	N/A
Alcian blue 8GX	Sangon Biotech	Cat# A600298
Alizarin Red S	Sangon Biotech	Cat# A506786
TRIzol Reagent	Life Technologies	Cat# 15596-018
RNA Nano 6000 Assay Kit	Agilent Technologies	Cat# 5067-1511
Hieff NGS Ultima Dual-mode mRNA Library Prep Kit for Illumina	Yeasen Biotechnology	Cat# 12309
RNA Isolater Total RNA Extraction Reagent	Vazyme	Cat# R401
HiScript III TR SuperMix for qPCR (+gDNA wiper) Reverse Transcription Kit	Vazyme	Cat# R323
SYBR qPCR Master Mix	Vazyme	Cat# Q712
Diaminobenzidine (DAB) staining solution	Pinuofei Biotechnology	Cat# PA3033

**Critical commercial assays**		

Mouse P1NP (Procollagen I N-Terminal Propeptide) ELISA Kit	Elabscience	Cat# E-EL-M0233
Mouse CTX1 (Cross Linked C-telopeptide of Type I Collagen) ELISA Kit	Elabscience	Cat# E-EL-M3023

**Deposited data**		

RNA-seq data	NCBI Sequence Read Archive (SRA)	BioProject: PRJNA1479650

**Experimental models: Organisms/strains**		

C57BL/6JGpt mice	GemPharmatech Co., Ltd	C57BL/6JGpt RRID: IMSR_GPT: N000013
Wild-type mice of the same genetic background	GemPharmatech Co., Ltd	WT; RRID: IMSR_GPT: N000013
cCNE300-KI mice	This paper	cCNE300-KI

**Oligonucleotides**		

sgRNAs targeting the CNE300 locus	This paper	Table S6
PCR genotyping primers	This paper	Table S6
RT-qPCR primers	This paper	Table S7

**Recombinant DNA**		

Donor vector containing the cetacean CNE300 sequence flanked by homology arms	This paper	N/A
Donor plasmid for cCNE300 knock-in	This paper	N/A

**Software and algorithms**		

LAS X (v 3.7.2)	Leica Microsystems	RRID:SCR_013673; https://www.leica-microsystems.com/products/microscope-software/p/leica-las-x-ls/
ImageJ (v 1.54r)	NIH	RRID: SCR_003070; http://imagej.org
NRecon (v 1.7.4.2)	Bruker, Kontic, Belgium	https://www.bruker.com/
CTAn (v 1.20.3.0)	Bruker, Kontic, Belgium	RRID:SCR_021338; https://www.bruker.com/en/products-and-solutions/preclinical-imaging/micro-ct/3d-suite-software.html
CTVox (v 3.3.0)	Bruker, Kontic, Belgium	https://www.bruker.com/
DataViewer (v 1.7.0)	Bruker, Kontic, Belgium	RRID:SCR_002633
SlideViewer (v 2.5.0)	3DHISTECH, Hungary	RRID:SCR_024885;https://www.3dhistech.com/
GraphPad Prism (v 8.2)	GraphPad Inc., San Diego, CA, USA	RRID:SCR_002798; www.graphpad.com
HISAT2 (v 2.0.4)	Kim et al.^34^ Daehwan Kim’s lab	RRID:SCR_015415 https://daehwankimlab.github.io/hisat2/
DESeq2 (v 1.30.1) R package	Love et al.^35^ Michael I. Love’s lab, UNC/HPH	RRID:SCR_015687 https://bioconductor.org/packages/DESeq2
clusterProfiler (v 4.4.4) R package	Wu et al.^36^ Guangchuang Yu’s lab	RRID:SCR_016481 https://bioconductor.org/packages/clusterProfiler

### Experimental model and study participant details

#### Animal

All animal experiments were conducted in strict accordance with the recommendations in the Regulations on the Management of Laboratory Animals of Nanjing Normal University. All procedures were reviewed and approved by the Animal Ethics and Welfare Committee of Nanjing Normal University (approval number: IACUC-20200501). All efforts were made to minimize the number of animals used and to reduce animal suffering throughout the experimental procedures.

The cetacean CNE300 knock-in (cCNE300-KI) mouse line was generated on a C57BL/6JGpt genetic background (RRID: IMSR_GPT: N000013). Wild-type (WT) littermates on the same genetic background were used as controls. All mice were housed under specific pathogen-free conditions at the NJNU Animal Resources Center (NJNU-ARC). The facility was maintained on a 12 h light/dark cycle (lights on at 7:00 a.m.) with controlled temperature (21 ± 1°C) and relative humidity (50–60%). Animals were provided with standard chow and water *ad libitum* and were monitored daily for health status and cage conditions in compliance with institutional guidelines.

Both male and female mice were used in this study, with only 1-week, 4-week and 12-week-old non-pregnant female mice used for morphometric analyses to minimize potential variability associated with sex differences. For developmental staging, female mice at 1-week, 4-week, and 12-week of age were randomly selected from litters and assigned to experimental groups. The sex and age of each animal are indicated in the corresponding figure legends. No formal statistical analysis of sex-associated effects was performed because the study was not designed to compare male and female phenotypes. For RNA-Seq experiments, the developmental stages of embryos were determined according to the criteria established by Lawson et al. (2016).^37^ Limb bud tissues were collected at E12.5 and E15.5 from embryos randomly selected from three independently pregnant WT and homozygous female mice, representing the early and late stage of limb bud development, respectively.

### Method details

#### CNE300 selection and sequence source

The conserved non-coding element 300 (CNE300, hg38: chr4:13,007,916-13,008,593) was selected for further *in vivo* functional validation based on previous comparative genomic and *in vitro* reporter assays.^21^ This element showed accelerated evolution in cetaceans and overlaps with a known human limb enhancer (hs259). In cetaceans, CNE300 contains lineage-specific sequence divergence, including a 11-bp deletion. Consistently, when the bottlenose dolphin CNE300 sequence was used as a representative cetacean sequence in dual-luciferase reporter assays, it showed significantly reduced regulatory activity performed at the cellular level. These prior findings supported the prioritization of CNE300 as a candidate regulatory element for *in vivo* functional analysis in the present study.

The cetacean orthologous CNE300 sequence used for generating the knock-in mouse model was derived from the bottlenose dolphin (*Tursiops truncatus*) genome assembly (Genome Warehouse: GWHBJYT00000000), Given its high sequence conservation across cetaceans and the high completeness and quality of the bottlenose dolphin genome, this species was selected as the representative cetacean sequence for functional characterization in this study. Full genomic coordinates and sequence information are provided in Table S6.

#### Generation of cCNE300-KI mice

The cCNE300-KI mouse model was generated using a CRISPR/Cas9-mediated genome editing strategy. Single guide RNAs (sgRNAs) targeting genomic regions flanking the intended insertion site were designed according to the experimental scheme and transcribed *in vitro*. A donor vector containing the *T. truncatus* CNE300 sequence flanked by homology arms was constructed. Cas9 mRNA (GemPharmatech Co., Ltd), sgRNAs, and the donor plasmid were co-injected into C57BL/6JGpt fertilized zygotes, which were subsequently transferred into pseudopregnant recipient females. Founder (F0) mice were identified by using PCR-based genotyping followed by Sanger sequencing, genetically confirmed F0 founders were crossed with WT mice of the same genetic background to obtain heterozygous F1 offspring. cCNE300-KI mice were then generated by intercrossing F1 heterozygotes. Primers used in this study were listed in Table S7. Exact sample sizes for each genotype and developmental stage are indicated in the corresponding figure legends.

#### Double staining and morphometric analyses

Female WT and cCNE300-KI mice at different development stages (1-week, 4-week, and 12-week) were randomly selected for double staining and morphometric analyses. and euthanized by CO_2_ asphyxiation. Prior to tissue processing, the individual’s body weight and body length (from nose tip to tail root) were measured, and then the skin and muscle tissues were removed, and the remaining samples were fixed overnight in 95% ethanol, followed by incubation in acetone for 48h. Skeletons were then stained with the Alcian blue 8GX (A600298, Sangon Biotech, Shanghai Co., Ltd.) and Alizarin red S (A506786, Sangon Biotech, Shanghai Co., Ltd.) solution at room temperature, as previously described.^38^ Subsequently, specimens were incubated in 1% NaOH to remove residual muscle tissue, then kept in glycerol for imaging using a Leica stereomicroscope (M165FC, Leica Microsystems Ltd., Wetzlar, Germany) and processed by Leica Application Suite X v3.7.2 (LAS X, Leica Microsystems Ltd., Wetzlar, Germany, RRID:SCR_013673). Images were measured using ImageJ v1.54r (RRID: SCR_003070, http://imagej.org) software. Relative body weight and length were normalized by dividing by the averages, and the relative lengths of limb long bones were calculated by normalizing individual bone lengths to the corresponding body length.

#### Micro-computerized tomography (CT) scanning

After the mice were sacrificed, the right limb long bones (humerus, femur, and tibia), cranium and spine of 12-week-old mice were isolated and fixed with 4% paraformaldehyde (PFA) for 48h and then maintained in 70% ethanol. Micro-CT scanning was performed with the SkyScan 1276 instrument (v1.8, Bruker, Kontic, Belgium) with the parameters of 60 kV (voltage) and 200 μA (current) at 6.0 μm voxel resolution. The images were reconstructed using the NRecon program v 1.7.4.2 (Bruker, Kontic, Belgium). The bone volume per tissue volume (BV/TV), bone mineral density (BMD), trabecular number (Tb.N), trabecular separation (Tb.Sp), trabecular thickness (Tb.Th) and trabecular pattern factor (Tb.Pf) were analyzed using the program CTAn v1.20.3.0 (Bruker, Kontic, Belgium, RRID:SCR_021338). Three-dimensional (3D) reconstructions were generated using CTVox v3.3.0 (Bruker, Kontic, Belgium), and the two-dimensional (2D) cross-sectional images were created by DataViewer v1.7.0 (Bruker, Kontic, Belgium, RRID:SCR_002633).

#### Histological staining and histomorphometric analyses

The excised limb bone samples were fixed in 4% PFA for 48h, followed by decalcification in 10% ethylenediaminetetraacetic acid (EDTA). Decalcified tissues were then dehydrated through graded ethanol and cleared in xylene. Subsequently, tissues were embedded in paraffin and sectioned at 5 μm. paraffin sections were subjected to hematoxylin and eosin (H&E) staining and tartrate-resistant acid phosphatase (TRAP) staining according to the standard protocols. Images were captured by PANNORAMIC scanner (3DHISTECH, Hungary), and SlideViewer v2.5.0 (3DHISTECH, Hungary, RRID:SCR_024885) software was used to select the delineate candidate regions, and TRAP-positive cells identified by red staining within these regions were quantified using ImageJ software. Statistical analyses were performed by using GraphPad v8.2 (GraphPad Inc., San Diego, CA, USA, RRID:SCR_002798) software.

#### RNA-seq analysis

Homozygous female mice that successfully conceived were selected to obtain embryos at E12.5 and E15.5. Forelimb and hindlimb bud tissues were collected, rapidly frozen in liquid nitrogen, and transferred to −80°C for subsequent experiments. The total RNA was extracted according to the instruction manual of the TRlzol Reagent (Cat# 15596-018, Life Technologies, California, USA). RNA concentration and purity was measured using NanoDrop 2000 (Thermo Fisher Scientific, Wilmington, DE). RNA integrity was assessed using the RNA Nano 6000 Assay Kit of the Agilent Bioanalyzer 2100 system (Cat# 5067-1511, Agilent Technologies, CA, USA).

Sequencing libraries were generated using Hieff NGS Ultima Dual-mode mRNA Library Prep Kit for Illumina (Cat# 12309, Yeasen Biotechnology (Shanghai) Co., Ltd.) following manufacturer’s recommendations and index codes were added to attribute sequences to each sample. The libraries were sequenced on an Illumina NovaSeq platform to generate 150 bp paired-end reads, according to the manufacturer’s instructions. The raw sequencing data have been deposited in the NCBI Sequence Read Archive (SRA) under BioProject accession number PRJNA1479650. The raw sequencing reads were processed using the BMKCloud online bioinformatics platform (www.biocloud.net).

Raw data (raw reads) of fastq format were firstly processed, and the high-quality clean reads were obtained after removing reads containing adapter, reads containing ploy-N and low-quality reads from raw data. Meanwhile, Q20, Q30, GC-content and sequence duplication level of the clean data were calculated. Hisat2 v2.0.4 tools (RRID:SCR_015415, https://daehwankimlab.github.io/hisat2/) were used to map with reference genome (mm10, GR Cm38).^34^ Gene function was annotated based on the following databases: Nr (NCBI non-redundant protein sequences), Pfam (Protein family), KOG/COG (Clusters of Orthologous Groups of proteins), Swiss-Prot (A manually annotated and reviewed protein sequence database), KEGG (The Kyoto Encyclopedia of Genes and Genomes), and GO (Gene Ontology). Quantification of gene expression levels were estimated by fragments per kilobase of transcript per million (FPKM) fragments mapped.

DESeq2 R package (RRID:SCR_015687, https://bioconductor.org/packages/DESeq2) was performed for Differential expression analysis.^35^ *p*-values were adjusted by the Benjamini and Hochberg’s approach for controlling the false discovery rate. Genes with an adjusted *p*-value <0.05 and Fold Change ≥2 found by DESeq2 were determined as differentially expressed genes (DEGs). Hierarchical cluster analysis of DEGs was further performed to show the gene expression pattern in two groups. GO and KEGG pathway enrichment analysis of DEGs were performed to elucidate the biological implications. GO enrichment was conducted using the clusterProfiler package v4.4.4 in R based on the Wallenius non-central hypergeometric distribution.^36^ KEGG pathway enrichment was assessed using the KOBAS database (http://kobas/cbi.pku.edu.cn/home.do) and clusterProfiler package v4.4.4 (RRID:SCR_016481, https://bioconductor.org/packages/clusterProfiler).

#### Reverse transcription quantitative PCR (RT-qPCR)

Total RNA was extracted using RNA Isolater Total RNA Extraction Reagent (R401, Vazyme, Nanjing, China) following the manufacturer’s protocols and reverse transcribed into cDNA using the HiScript III TR SuperMix for qPCR (+gDNA wiper) Reverse Transcription Kit (R323, Vazyme, Nanjing, China). qPCR was performed using SYBR qPCR Master Mix (Q712, Vazyme, Nanjing, China) with specific primers (Table S8) on a Roche LightCycler® 480 system (Roche Diagnostics, Basel, Switzerland). Relative gene expression was calculated using the 2^−ΔΔct^ method.^39^

#### Immunohistochemical (IHC) staining

For IHC staining, bone sections were prepared as described above. Sections were dewaxed, rehydrated, and subjected to antigen retrieval using 2× citrate buffer (pH 6.0), endogenous peroxidase activity was quenched with 3% hydrogen peroxide, then blocked with 10% goat serum at room temperature for 30 min. Primary antibodies RUNX2 (A2851, Abclonal, Wuhan, China, 1:200) were incubated overnight at 4°C. After washing with phosphate-buffered saline containing 0.1% Tween 20 (PBST), sections were incubated with an HRP-conjugated goat anti-ribbit IgG secondary antibody (PN0046, Pinuofei Biotechnology, Wuhan, China, 1:1000 in PBST) for 1h at 37°C, followed by three additional PBST washes. Diaminobenzidine (DAB) (PA3033, Pinuofei Biotechnology, Wuhan, China) staining was performed, and nuclei were counterstained with hematoxylin. Finally, sections were dehydrated, cleared and mounted.

#### Enzyme-linked immunosorbent assay (ELISA)

Blood samples were collected from 12-week-old female mice, allowed to clot at room temperature for 1h, and were subsequently centrifuged at 1000× g for 20 min to isolate serum. The level of Procollagen Ⅰ N-Terminal Propeptide (PINP) and Cross-Linked C-telopeptide of Type Ⅰ Collagen (CTX-1) were detected using the Mouse P1NP ELISA Kit (E-EL-M0233, Elabscience, Wuhan, China) and Mouse CTX-1 ELISA Kit (E-EL-M3023, Elabscience, Wuhan, China), respectively, according to the manufacturer’s instructions.

### Quantification and statistical analysis

All data were presented as mean ± standard deviation (mean ± SD). Exact sample sizes for each experimental group are indicated in the corresponding figure legends. Unless otherwise stated, n represents the number of biologically independent animals or embryos. For graphs showing individual data points, each dot represents one biologically independent sample. Representative images are shown from biologically independent samples. Statistical analyses were performed using GraphPad Prism v8.2. Data normality was assessed using the Shapiro–Wilk test. For comparisons between two groups, an unpaired Student’s *t* test was applied for normally distributed data, whereas the Mann–Whitney U test was used otherwise. Statistical significance was denoted as follows: ∗*p* < 0.05; ∗∗*p* < 0.01; ∗∗∗*p* < 0.001; ns, no significant.

## Supplemental information

Document S1. Figures S1–S5

Table S1. Morphometric measurements for WT and cCNE300-KI mice, related to Figure 1

Table S2. Quantitative measurements of limb skeletal parameters in WT and cCNE300-KI mice, related to Figure 2

Table S3. Quantitative measurements of skull and spine parameters in WT and cCNE300-KI mice, related to Figure S3

Table S4. DEGs of the cCNE300-KI mice compared with the WT embryos at E12.5, related to Figure 4

Table S5. DEGs of the cCNE300-KI mice compared with the WT embryos at E15.5, related to Figure 4

Table S6. Genomic coordinates and sequence information of CNE300 used in this study, related to STAR Methods

Table S7. Primer sequences information in this study, related to STAR Methods

Table S8. Primers used for qPCR amplification in this study, related to STAR Methods
