# Supplementary material for: A cetacean-specific CNE suppresses osteogenesis to drive appendicular modification in secondary aquatic adaptation
Source: iScience. 2026 Sep 3;29(9):117145. doi: 10.1016/j.isci.2026.117145 (PMC13571659; doi:10.1016/j.isci.2026.117145)
Supplement: Supplementary file 1 [file mmc1.pdf]

iScience, Volume *29*

## Supplemental information

# A cetacean-specific CNE suppresses osteogenesis to drive appendicular modification in secondary aquatic adaptation

**Yao Liu, Zhenhua Zhang, Fenli Zhang, Shixia Xu, and Guang Yang**

**Supplemental information**

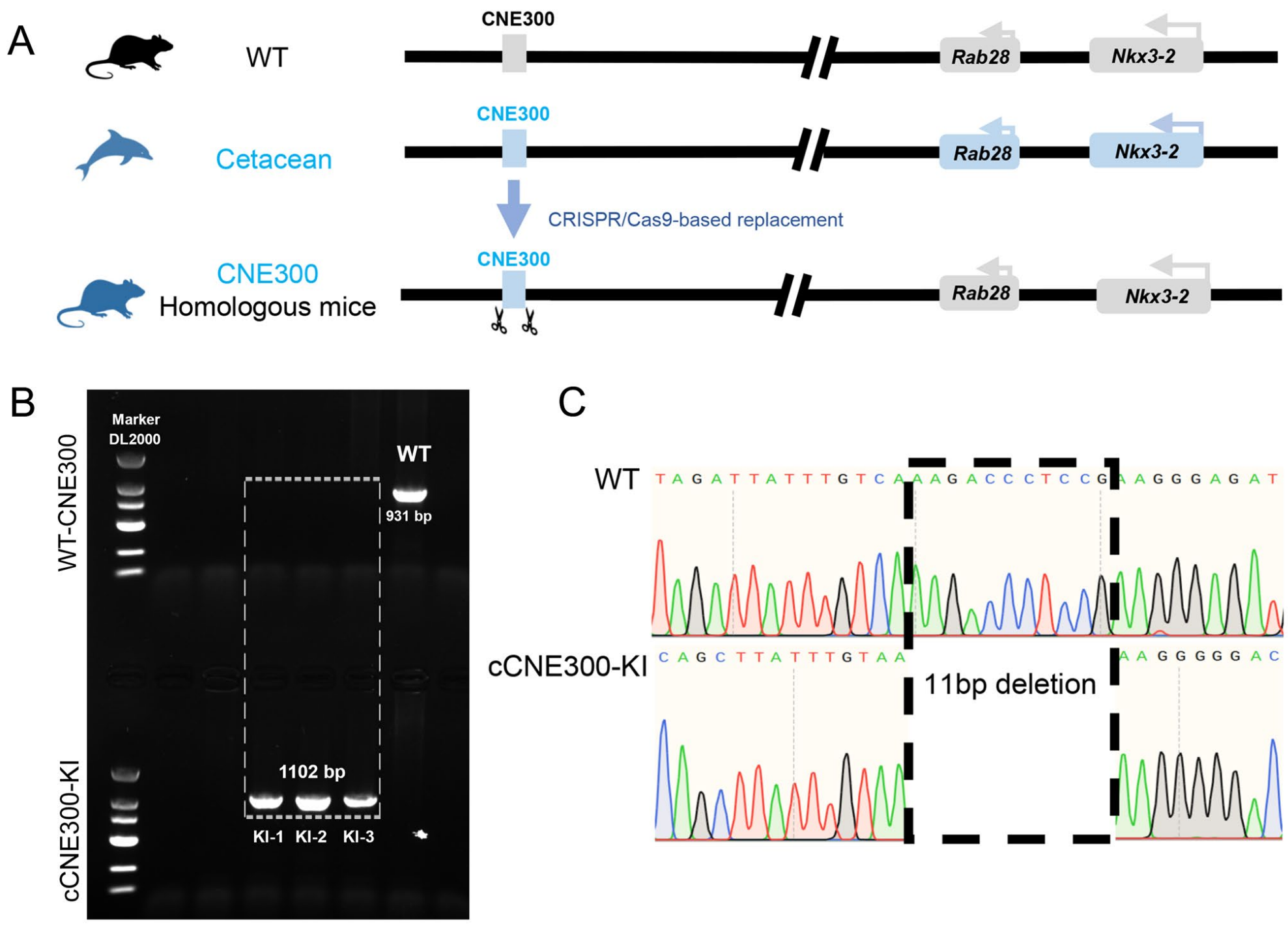


**Figure S1. Generation and validation of the cetacean CNE300 homozygous knock-in mouse model. Related to STAR Methods.** (A) Schematic of generating a cetacean CNE300 homologous replacement mouse model by CRISPR/Cas9. (B) Agarose gel electrophoresis plot of genotyping for PCR validation of the CNE300 sequences by designed specific primers. Lanes KI-1, KI-2, KI-3 (1102 bp) display a single lower band, indicating homozygous mice, whereas lane WT1 (931 bp) corresponds to the WT control. (C) Sanger sequencing of the WT and cetacean CNE300 sequences, the full cetacean CNE300 with 11bp deletions were successfully in the cCNE300-KI genome.

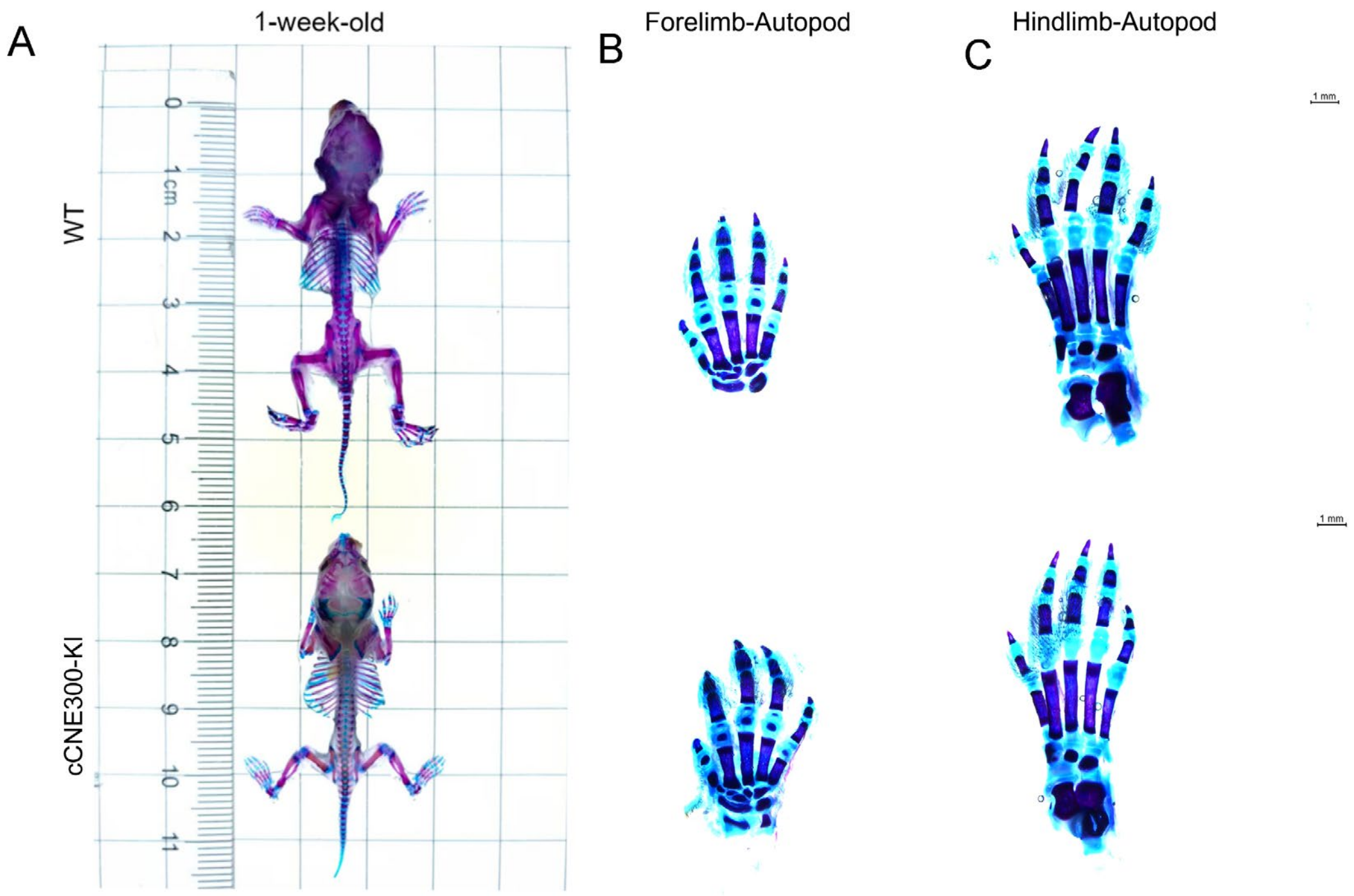


**Figure S2. cCNE300-KI mice exhibit normal skeletal integrity and digit numbers in distal appendages. Related to Figure 1B.** (A) Representative Alcian blue and Alizarin red double staining of the whole skeleton from 1-week-old WT and cCNE300-KI mice. (B, C) Magnified views of the forelimb (B) and hindlimb (C) autopods, showing intact metacarpals, metatarsals, and phalanges. No obvious differences in skeletal integrity or digit numbers were observed between the two genotypes. Scale bars, 1 mm.

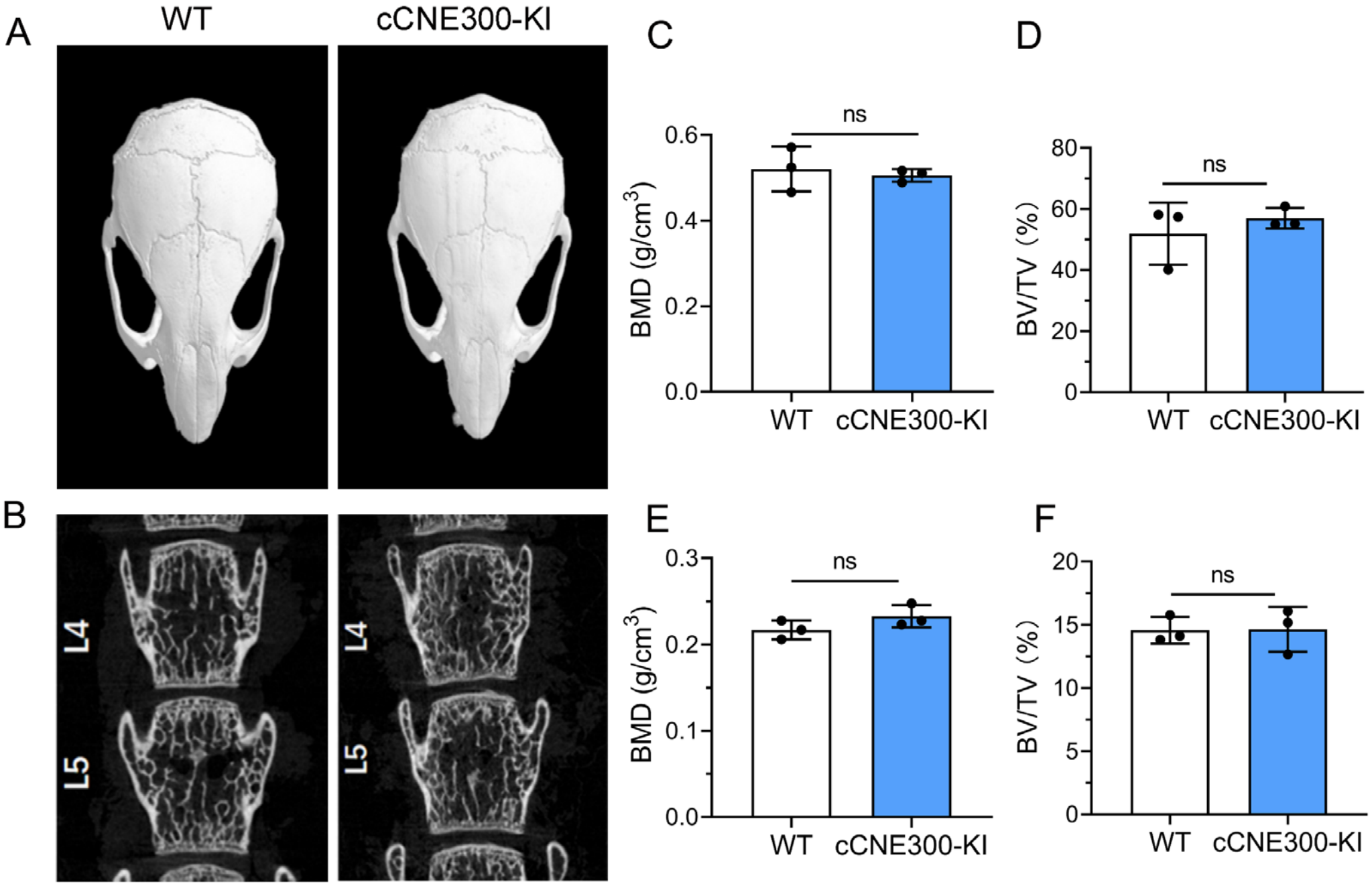


**Figure S3. cCNE300-KI mice show no alterations in skull and vertebral bone microarchitecture, related to "Cetacean CNE300 alters limb skeletal microarchitecture and reduces trabecular bone mass" section.** (A, B) Representative micro-CT 3D reconstructions of the skull (A) and coronal views of the spine L4 and L5 (B) from WT and cCNE300-KI mice. Scale bars, 100 μm. (C, D) Quantitative analyses of bone mineral density (BMD) (C), and bone volume fraction (BV/TV) (D) of the skull. (E, F) Quantitative analyses of BMD (E) and BV/TV (F) of the spine L5. In the bar charts, each dot represents an individual mouse (n = 3). Data are presented as mean ± SD. Student's *t*-test, ns, not significant. See also Table S3.

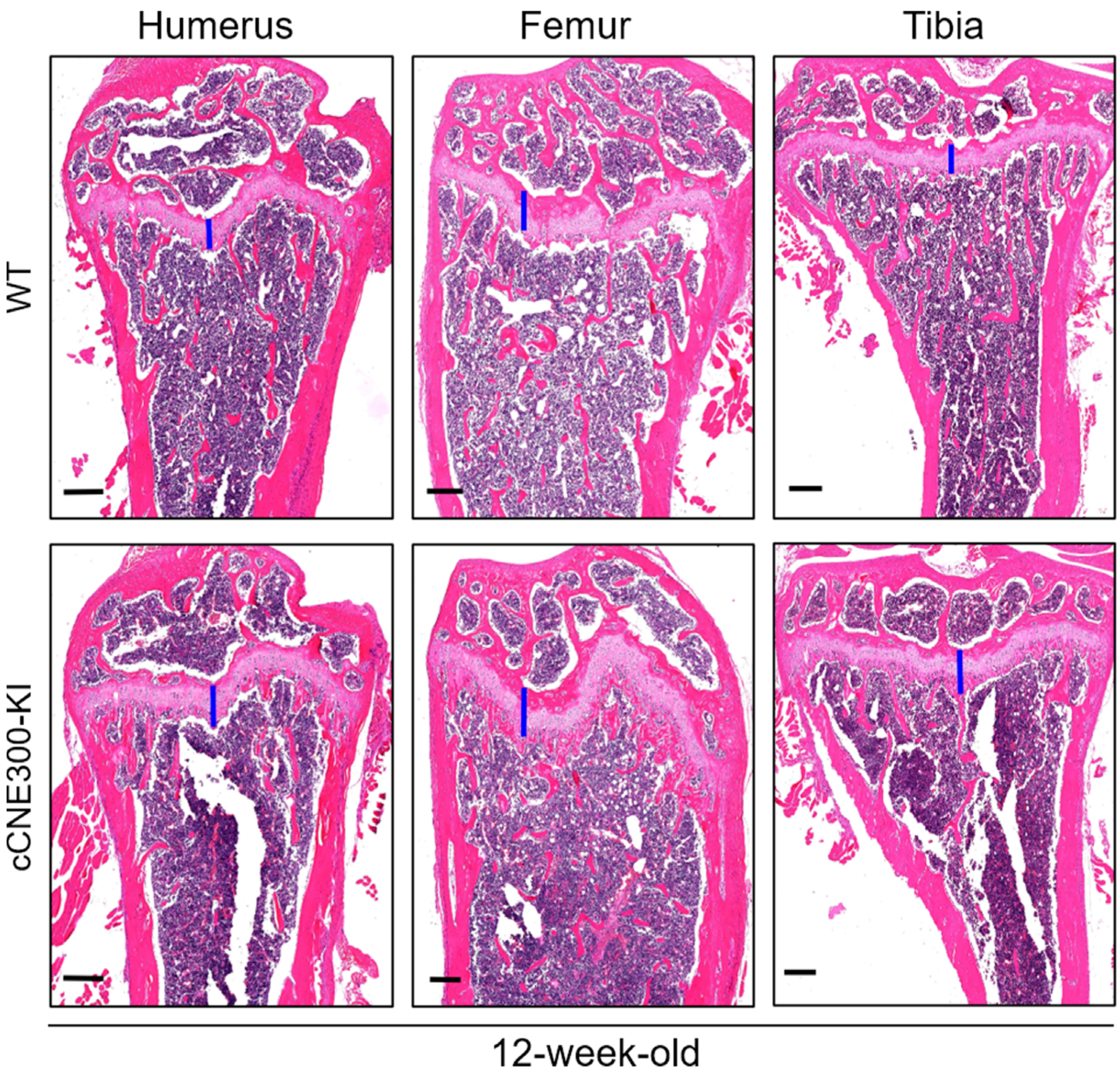


**Figure S4. Representative images of H&E staining of limb bone sections of WT and cCNE300-KI mice with the indicated ages, related to Figure 3.** scale bars, 250 μm. growth plate is indicated in blue.

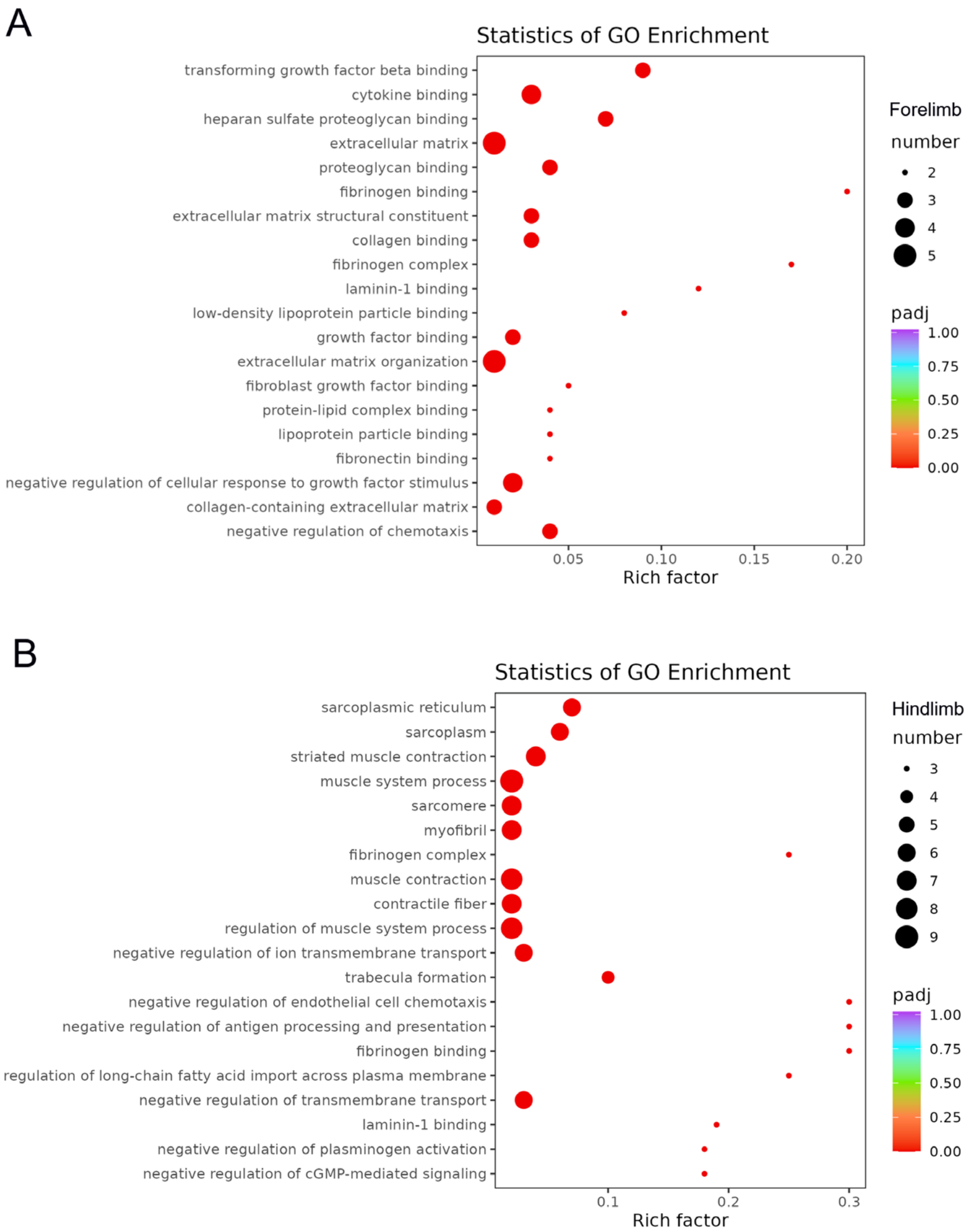


**Figure S5. GO enrichment of DEGs in E12.5 limb buds, related to Figure 4A.** (A) GO enrichment analysis of DEGs in forelimb buds. (B) GO enrichment analysis of DEGs in Hindlimb buds.
